# Nuage condensates: accelerators or circuit breakers for sRNA silencing pathways?

**DOI:** 10.1261/rna.079003.121

**Published:** 2022-01

**Authors:** John Paul Tsu Ouyang, Geraldine Seydoux

**Affiliations:** HHMI and Department of Molecular Biology and Genetics, Johns Hopkins University School of Medicine, Baltimore, Maryland 21205, USA

**Keywords:** nuage, condensate, small RNA, Argonaute, RNAi, piRNAs, small RNA amplification

## Abstract

Nuage are RNA-rich condensates that assemble around the nuclei of developing germ cells. Many proteins required for the biogenesis and function of silencing small RNAs (sRNAs) enrich in nuage, and it is often assumed that nuage is the cellular site where sRNAs are synthesized and encounter target transcripts for silencing. Using *C. elegans* as a model, we examine the complex multicondensate architecture of nuage and review evidence for compartmentalization of silencing pathways. We consider the possibility that nuage condensates balance the activity of competing sRNA pathways and serve to limit, rather than enhance, sRNA amplification to protect transcripts from dangerous runaway silencing.

## INTRODUCTION

In eukaryotic cells, transcription and translation occur in separate compartments, and mRNAs must be exported from the nucleus for translation in the cytoplasm. In somatic cells, mRNAs immediately encounter ribosomes upon exit from the nucleus. In developing germ cells, the journey involves an extra step: mRNAs traverse a perinuclear compartment called nuage before reaching the cytoplasm. Nuage is a conserved feature of germ cells in metazoans and was first identified using electron microscopy as a collection of electron-dense, amorphous “clouds” surrounding germ cell nuclei (Eddy 1975; Voronina et al. 2011). We now know that nuage is a collection of membraneless condensates rich in RNA and RNA-binding proteins. Many proteins in nuage are implicated in RNA-mediated interference, and it is often assumed that silencing small RNAs are synthesized in nuage.

In this review, we use the *C. elegans* model to examine our current understanding of nuage structure and function. We begin with a brief introduction to sRNA pathways in *C. elegans* and a description of the cell biology of nuage. We review evidence that implicates nuage condensates in sRNA amplification and activity, as well as evidence that challenges the hypothesis that nuage is an obligate compartment for sRNA amplification. Finally, we consider a unifying model where nuage condensates limit, rather than enhance, sRNA amplification to prevent dangerous runaway loops.

## SMALL RNA PATHWAYS IN *C. ELEGANS*

**FIGURE 1.**
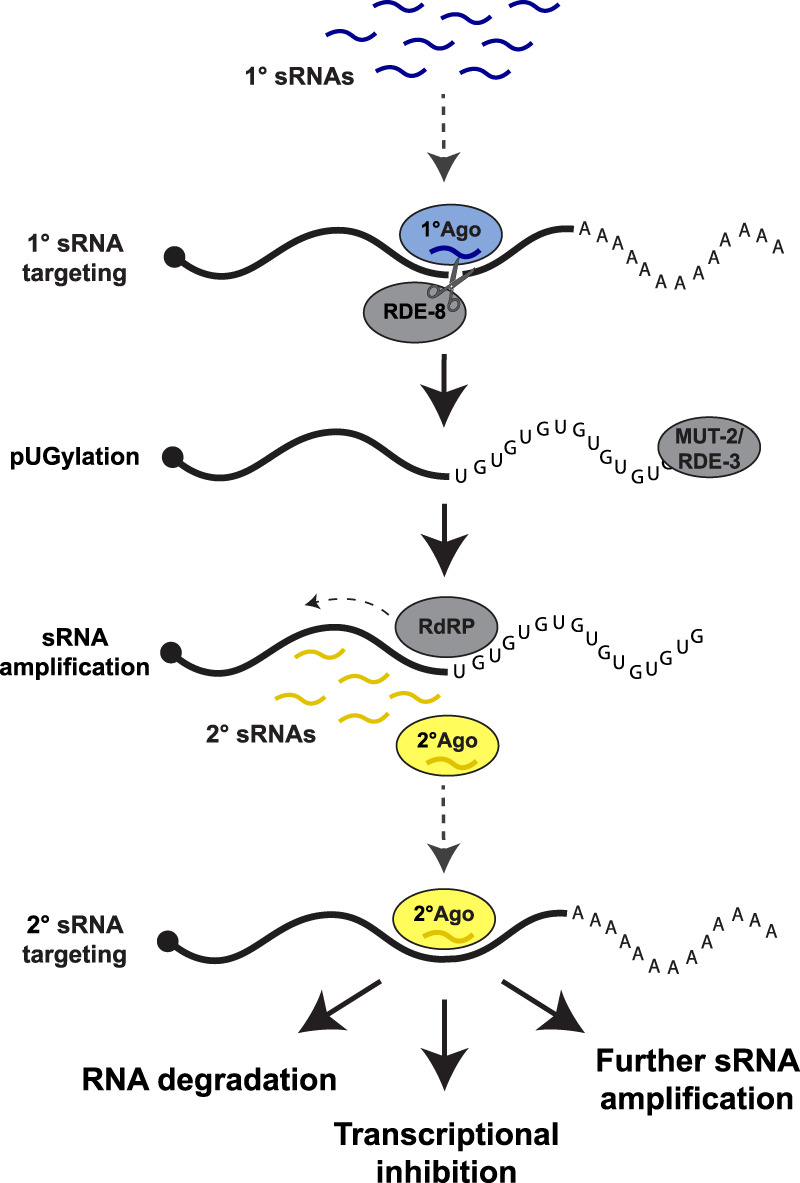
Silencing by sRNAs in *C. elegans*. Primary sRNAs (blue wavy lines) bind to primary Argonaute proteins (blue ovals) and target complementary transcripts for cleavage by RDE-8 and “pUGylation” by the pUGylase MUT-2/RDE-3. The pUG tail recruits RNA-dependent RNA polymerases (RdRPs) that use the RNA fragment as a template for synthesis of secondary sRNAs antisense to the pUGylated transcript. Secondary sRNAs bind to secondary Argonautes (yellow) and target complementary transcripts for degradation, transcriptional inhibition, or additional rounds of sRNA amplification. Dotted arrows indicate a sRNA targeting event.

sRNAs are regulatory RNAs, 20–30 nt in length, that associate with a class of proteins known as Argonautes. In *C. elegans*, there are two general categories of sRNAs: primary sRNAs and secondary sRNAs (Fig. 1). Primary sRNAs originate from genomically encoded transcripts (e.g., “piRNAs”), from RNA-dependent RNA polymerases (RdRPs) that target particular mRNAs (e.g., 26G-sRNAs), or from Dicer-mediated processing of double-stranded RNAs derived from endogenous or exogenous sources (including bacteria engineered to produce double-stranded RNA in “feeding RNAi” experiments). Primary sRNA/Argonaute complexes recognize cognate mRNAs through base-pair complementarity (Fig. 1). Upon recognition by primary sRNA/Argonaute complexes, transcripts are cleaved by the endonuclease RDE-8 (Tsai et al. 2015) and tailed by the poly(UG) polymerase MUT-2/RDE-3 (“pUGylation,” Shukla et al. 2020). pUG tails recruit RdRPs that synthesize “secondary” sRNAs complementary to the pUGylated transcripts (22G-RNAs; Shukla et al. 2020). This step serves to amplify the pool of sRNAs against the targeted RNA. Secondary sRNAs associate with “secondary Argonautes.” The worm genome encodes 19 functional Argonautes, 12 of which are thought to associate with amplified secondary sRNAs (Yigit et al. 2006; Sundby et al. 2021). Depending on the Argonaute, secondary sRNAs target cognate mRNAs in the cytoplasm for degradation (Yigit et al. 2006; Gu et al. 2009) or nascent transcripts in nuclei to initiate chromatin-based silencing of the locus (Guang et al. 2008; Burkhart et al. 2011; Burton et al. 2011; Buckley et al. 2012; Gu et al. 2012). Secondary sRNAs inherited by progeny can also restart the pUGylation amplification cycle to generate more secondary sRNAs (Shukla et al. 2020). Amplification cycles initiated by secondary sRNAs continue even in the absence of primary sRNA triggers, allowing for the transmission of secondary sRNAs from parent to progeny in a process known as RNAi inheritance (Fire et al. 1998; Grishok et al. 2000; Alcazar et al. 2008; Ashe et al. 2012; Shirayama et al. 2012).

sRNAs are typically associated with silencing, but there also exists a class of sRNAs that target transcripts that are robustly expressed in the germline. CSR-1 sRNAs (so named because of their association with the Argonaute CSR-1) are synthesized by an RdRP that uses germline-expressed transcripts as templates in a process that is not well understood (Billi et al. 2014; Weiser and Kim 2019; Reed et al. 2020). CSR-1 sRNAs have been proposed to protect germline transcripts from piRNA-induced silencing. The *C. elegans* genome encodes over 15,000 piRNAs with sufficient sequence diversity to theoretically silence the entire *C. elegans* transcriptome (Bagijn et al. 2012; Lee et al. 2012; Shen et al. 2018; Zhang et al. 2018). By opposing piRNA silencing, CSR-1 sRNAs have been proposed to “license” genuine germline transcripts for expression (Seth et al. 2013; Wedeles et al. 2013; Shen et al. 2018). CSR-1 has also been shown to use its slicer activity to fine-tune the levels of certain transcripts coding for maternal proteins active in embryos (Gerson-Gurwitz et al. 2016; Quarato et al. 2021).

## CELL BIOLOGY OF NUAGE

**FIGURE 2.**
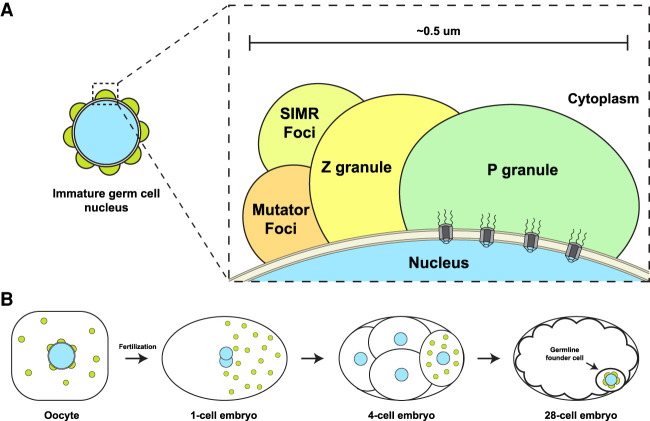
Nuage compartments. (*A*) Nuage are perinuclear condensates adsorbed to the cytoplasmic face of the nucleus in immature germ cells. Nuage contains at least four distinct condensates that enrich different components of the sRNA machinery. The exact orientation of nuage condensates relative to each other and the nucleus is not known and varies depending on developmental stage. (*B*) In transcriptionally quiescent oocytes, Mutator and SIMR foci disassemble (not shown), and P and Z granules merge and relocalize to the cytoplasm with other cytoplasmic condensates to form large assemblies called germ granules (green circles). Germ granules segregate to the posterior of zygotes for segregation to the germline. Distinct Mutator, SIMR, P, and Z condensates re-form in the germline founder cell.

As in other organisms (Czołowska 1969; Strome and Wood 1982; Kloc et al. 2004; Chuma et al. 2009), nuage in *C. elegans* is most prominent in transcriptionally active, immature germ cells in early stages of meiosis (pachytene). By electron microscopy, nuage condensates appear as half-moons spread over nucleopore-rich regions of the nuclear envelope (Fig. 2). Pitt et al. (2000) have estimated that 75% of nucleopores in immature germ cells are covered by nuage. Nuage stains strongly with RNA dyes and probes (Pitt et al. 2000; Schisa et al. 2001; Sheth et al. 2010). Visualization of newly transcribed RNAs by [^3^H]uridine pulse labeling and by in situ hybridization confirmed that newly synthesized transcripts enrich in nuage upon exit from the nucleus (Schisa et al. 2001; Sheth et al. 2010). The transcriptional inhibitor α-amanitin causes the nuage proteins PGL-1 and MUT-16 to disperse in the cytoplasm, suggesting that newly transcribed RNAs are an integral component of nuage (Sheth et al. 2010; Uebel et al. 2020). Nuage components also disperse in the cytoplasm when germ cells mature into transcriptionally silent gametes. In oocytes, a subset of nuage components associate with other condensates to form complex assemblies (“germ granules”) in the cytoplasm (Updike and Strome 2010; Seydoux 2018; Marnik and Updike 2019). After fertilization, germ granules become asymmetrically localized in the cytoplasm for segregation to the germline founder cells of the embryo (Fig. 2B). When transcription restarts in these cells, nuage condensates reassemble at the nuclear membrane (Updike and Strome 2010; Uebel et al. 2020, 2021). The continuity of nuage during development suggests that most, if not all, germline transcripts experience nuage before entering the bulk cytoplasm.

Nuage is not a homogenous structure but rather a collection of distinct condensates. By electron microscopy, nuage contains subdomains of differing electron density including a dense layer closest to the nuclear envelope and dense “crests” facing the cytoplasm (Sheth et al. 2010). Localization of nuage proteins by fluorescence microscopy has confirmed that nuage is a layered structure containing at least four condensate classes each defined by two or more unique components (Fig. 2A): SIMR foci, Mutator foci, P granules, and Z granules (for review, see Sundby et al. 2021). SIMR and Mutator foci are adjacent (Manage et al. 2020), and Z granules reside between P granules and SIMR/Mutator foci (Fig 2A; Wan et al. 2018; Manage et al. 2020). Interestingly, the spatial organization of nuage evolves over developmental time (Wan et al. 2018; Toraason et al. 2021). For example, markers for P granules and Z granules overlap in a subset of nuage assemblies in immature germ cells and merge completely in the cytoplasmic germ granules of early embryos (Wan et al. 2018; Toraason et al. 2021). P and Z granules de-mix again when nuage condensates re-form around the nuclei of the embryonic germline founder cell (Wan et al. 2018).

## LIQUID-LIKE PROPERTIES OF NUAGE CONDENSATES

Nuage condensates are thought to form by liquid–liquid phase separation, a thermodynamic process that drives condensation of interacting polymers into dense droplets (Brangwynne et al. 2009; Hyman et al. 2014; Seydoux 2018; Dodson and Kennedy 2020). Weak, multivalent protein:protein and protein:RNA interactions create large interaction networks that drive phase separation while maintaining molecules in a dynamic state, free to diffuse within the condensate and to exchange with the cytoplasm. Liquid-like behavior has been most thoroughly documented for the P granule subdomain of nuage. P granule proteins exchange rapidly between nuage and cytoplasm and disperse into the cytoplasm when exposed to high temperatures (Brangwynne et al. 2009; Putnam et al. 2019; Fritsch et al. 2021). P granules also fuse and “drip off” nuclei when subjected to shear forces (Brangwynne et al. 2009).

P granules are assembled by the VASA-related GLH helicases and RGG domain-containing PGL proteins (Updike and Strome 2010). GLH helicases contain FG repeats also found in nucleoporins that line the central channel of nucleopores (Updike et al. 2011; Marnik et al. 2019). FG nucleoporins form a matrix that functions as the nucleus’ permeability barrier (Beck and Hurt 2017). P granules exhibit similar permeability properties and have been proposed to function as extensions of the nucleopore environment (Updike et al. 2011). PGL proteins contain two dimerization domains and RNA-binding RGG domains (Aoki et al. 2016, 2021). Purified PGL-3 forms condensates in vitro in a manner that is stimulated by RNA, consistent with the apparent RNA requirement for P granule integrity in vivo (Saha et al. 2016; Putnam et al. 2019).

Other nuage condensates have also been reported to exhibit liquid-like behavior. Fluorescently labeled proteins in Z granules and Mutator foci exhibit rapid recovery after photobleaching, consistent with rapid exchange with a cytoplasmic pool (Uebel et al. 2018; Wan et al. 2018). Mutator foci components dissolve when exposed to high temperatures and have been shown to form in a manner dependent on the concentration of the core scaffolding component MUT-16 (Phillips et al. 2012; Uebel et al. 2018). While a core Z granule protein has not been identified, Z granule morphology appears to depend on the piRNA biogenesis factor PID-2/ZSP-1 (Placentino et al. 2021; Wan et al. 2021). Super-resolution microscopy has revealed that PID-2/ZSP-1 localizes to the outside surface of Z granules (Wan et al. 2021), and mutants lacking PID-2/ZSP1 have enlarged Z granules (Placentino et al. 2021; Wan et al. 2021). Less is known about the underling formation and properties of SIMR foci.

What causes nuage condensates to contact each other while maintaining distinct compositions? Reconstitution experiments in human cells suggest that competition between overlapping interaction networks tune the segregation of select proteins into P bodies or stress granules. Interaction nodes that connect the competing networks cause P bodies and stress granules to “wet”—come in close contact—with each other (Sanders et al. 2020). Similar principles could potentially apply to nuage, since interactions between proteins enriched in different condensates have been reported. For example, the Z granule protein ZNFX-1 coimmunoprecipitates with the P granule proteins EGO-1, PRG-1, and CSR-1 (Ishidate et al. 2018; Barucci et al. 2020). Similarly, the SIMR granule protein SIMR-1 was identified through mass spectrometry analyses of MUT-16 complexes (Manage et al. 2020). In principle, developmentally regulated changes in protein–protein interactions could tune the overlap between competing networks, leading to condensate mixing and de-mixing over developmental time, as observed for Z and P granules during oogenesis and early embryogenesis.

## COMPARTMENTALIZATION OF THE sRNA MACHINERY IN NUAGE CONDENSATES

Functional modules in the sRNA machinery segregate to different condensates in nuage (Table 1). Six Argonautes, including the competing Argonautes PRG-1 and CSR-1, enrich in P granules (Batista et al. 2008; Claycomb et al. 2009; Gu et al. 2009; Conine et al. 2010; Gerson-Gurwitz et al. 2016; Brown et al. 2017). Consequently, P granules have been proposed to function as the principal site for transcript recognition by Argonautes, allowing Argonautes to survey every germline transcript immediately upon exiting the nucleus (Sundby et al. 2021). In contrast, components of the sRNA amplification machinery, including the endonuclease RDE-8, the pUGylase MUT-2/RDE-3, and the RdRP RRF-1, localize to Mutator foci (Phillips et al. 2012; Tsai et al. 2015; Uebel et al. 2018). Genetic loss of Mutator foci components, including the core scaffold MUT-16, prevents the production of secondary sRNAs (Zhang et al. 2011; Phillips et al. 2014). Mutator foci, therefore, have been proposed to function as centers for sRNA amplification (Dodson and Kennedy 2020; Sundby et al. 2021). Other factors required for sRNA amplification, however, enrich in condensates adjacent to mutator foci. The Tudor domain protein SIMR-1, required for amplification of sRNAs downstream from endogenous piRNAs, is the defining protein for SIMR foci (Manage et al. 2020). Lastly, the helicase ZNFX-1 and the Argonaute WAGO-4 (Wan et al. 2018), required for RNAi inheritance, enrich in Z granules (Ishidate et al. 2018; Wan et al. 2018; Xu et al. 2018). ZNFX-1 interacts with the RdRP EGO-1 (Ishidate et al. 2018), suggesting that Z granules also function in sRNA amplification. These localizations suggest that several nuage condensates specialize in sRNA amplification. In the next section, we review evidence in support of this “guilt by association” hypothesis.

**TABLE 1.**
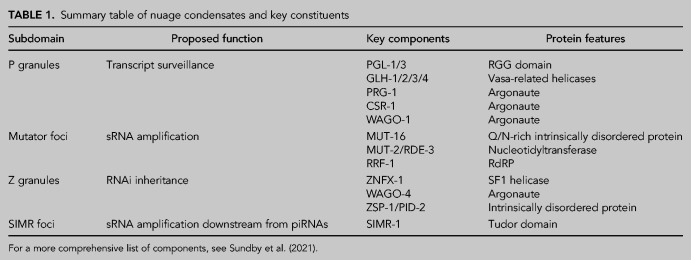
Summary table of nuage condensates and key constituents

## EVIDENCE LINKING NUAGE TO sRNA FUNCTION

A common hypothesis is that the unique material properties of nuage condensates create a favorable biochemical environment for sRNA biogenesis and/or recognition of target mRNAs by sRNA/Argonaute complexes (Phillips et al. 2012; Dodson and Kennedy 2020; Sundby et al. 2021). Transcripts recognized by Argonautes in P granules are hypothesized to be funneled to other nuage condensates for sRNA amplification. The dynamic liquid-like environment of nuage could theoretically accelerate amplification by enabling the fast exchange of intermediates between neighboring nuage condensates. Support for this model has come from examining the localization of transcripts targeted by RNAi. In situ hybridization experiments have shown that transcripts targeted by feeding RNAi, as well as pUGylated transcripts, accumulate in nuage (Shukla et al. 2020; Ouyang et al. 2021). Although the resolution of in situ hybridization experiments makes the identification of the exact condensate difficult, RNAi-targeted transcripts were found to overlap with P granule and Z granule markers (Ouyang et al. 2021), and pUGylated transcripts overlapped with Mutator foci (Shukla et al. 2020). Additional evidence for a role for P granules in transcript silencing has come from elegant experiments using a GFP reporter transcript artificially tethered to the P granule protein PGL-1. The tethered transcript was silenced in a manner dependent on P granule assembly and the Argonaute WAGO-1, consistent with a sRNA-dependent mechanism (Aoki et al. 2021). Other studies have shown that disassembly of P granules by knocking down key P granule scaffolds leads to accumulation of transcripts normally expressed in somatic and sperm cells (Updike et al. 2014; Knutson et al. 2017). These studies have cemented the hypothesis that P granules function as triage centers where genuine germline transcripts are separated from unwanted transcripts that need to be silenced.

## sRNA MACHINERY IS ALSO ACTIVE IN THE CYTOPLASM?

The liquid-like properties of nuage condensates indicate that nuage components are constantly exchanging between nuage and the cytoplasm. Germ cells share a common cytoplasm (rachis) that runs through the entire germline; the relative proportion of nuage molecules in the bulk cytoplasm versus nuage is not known but is likely to be significant since the bulk cytoplasm occupies a much larger volume. A systematic study in yeast revealed that, even for proteins that are highly enriched in P bodies, 70% of molecules are in the cytoplasm (Xing et al. 2020). We cannot discount, therefore, the possibility that a significant proportion of nuage proteins are present and active in the cytoplasm. Several lines of evidence in fact support this view. First, sRNA amplification has been observed in homogenized *C. elegans* lysates, suggesting that this process can function outside of condensates (Aoki et al. 2007). Second, sRNA amplification also occurs robustly in somatic cells, where the sRNA machinery does not visibly concentrate in condensate structures (Phillips et al. 2012). Third, levels of pUGylated transcripts produced in response to feeding RNAi increase in *mut-16* mutants, which do not assemble Mutator condensates (Shukla et al. 2020). Fourth, in animals lacking the helicase ZNFX-1, mRNAs targeted by RNAi no longer accumulate in nuage, yet are still subjected to robust pUGylation, sRNA amplification, and degradation, presumably in the cytoplasm (Ouyang et al. 2021). Finally, mutants that prevent nuage assembly in embryonic germline founder cells cause an increase, rather than decrease, in sRNA amplification leading to the inappropriate silencing of endogenous transcripts (Dodson and Kennedy 2019; Lev et al. 2019; Ouyang et al. 2019). In *meg-3 meg-4* mutants, condensation of nuage proteins is delayed in the germline founder cells (Wang et al. 2014). The first transcripts expressed in these cells are released directly into the cytoplasm (Ouyang et al. 2019). Transcripts targeted by piRNAs become hyper-targeted by secondary sRNAs and silenced in *meg-3 meg-4* mutants, presumably because they are no longer protected by CSR-1 in the P granule environment (Ouyang et al. 2019). Similarly, silencing initiated by feeding RNAi can be perpetuated for more generations in *meg-3 meg-4* mutants compared to wild-type animals (Lev et al. 2019). Together, these findings suggest that condensation of nuage proteins restrain, rather than promote, sRNA amplification.

## HYPOTHESIS: SPATIAL SEGREGATION OF THE sRNA MACHINERY ACROSS MULTIPLE NUAGE CONDENSATES PROTECTS GERM CELLS FROM DANGEROUS AMPLIFICATION LOOPS

Reiterative cycles of sRNA amplification pose serious risks to organisms as theoretically they could lead to irreversible silencing of endogenous genes. Pak et al. (2012) showed that such dangerous feedback loops are avoided in somatic cells by allowing only primary sRNAs to initiate sRNA amplification. In somatic cells, secondary sRNA/Argonaute complexes cause degradation of targeted transcripts and are not allowed to engage with RNA-dependent RNA polymerases. Sapetschnig et al. (2015) showed that, in contrast, in the germline, secondary sRNA/Argonaute complexes are permitted to initiate sRNA amplification, leading to the production of “tertiary sRNAs.” This ability is essential to perpetuate silencing from mother to progeny (RNAi inheritance). Breaks on transgenerational inheritance, however, must also exist as gene silencing initiated by feeding RNAi typically only lasts for four to five generations (Rechavi and Lev 2017).

**FIGURE 3.**
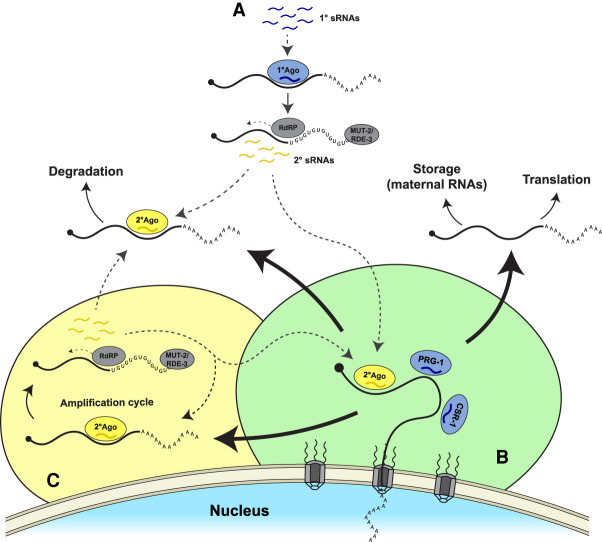
Working model for functional compartmentalization of sRNA pathways. Dotted arrows link sRNA production sites to sites where sRNAs/Argonautes target transcripts. Continuous arrows denote evolution of mRNAs. (*A*) In the cytoplasm, primary sRNAs associate with primary Argonautes (blue), leading to pUGylation, and synthesis of secondary sRNAs (yellow). Secondary sRNA/Argonaute complexes target complementary mRNAs for degradation in the cytoplasm or target transcripts emerging from the nucleus (see below). (*B*) Upon exit from the nucleus, newly transcribed mRNAs are “read” by competing silencing and nonsilencing Argonautes in “triage condensates” (likely to correspond to P granules). mRNAs are then sent off to three possible fates: (i) translation or storage in the cytoplasm, (ii) degradation in the cytoplasm, or (iii) use as templates for secondary sRNA amplification in other nuage condensates (SIMR, Mutator foci or Z granules) that house self-perpetuating amplification loops initiated by secondary sRNAs (*C*).

We hypothesize that compartmentalization of the sRNA machinery in nuage serves to prevent dangerous runaway sRNA amplification loops by sequestering different biochemical activities into different compartments (Fig. 3). In particular, we propose that concentration of Argonautes in P granules away from sRNA amplification centers in other condensates increases the stringency by which transcripts are screened before silencing. Juxtaposition of competing licensing and silencing Argonautes in P granules could create a “silencing rheostat” that precisely tunes the number of RNA molecules for each transcript that are shuttled directly to cytoplasm or allowed to visit other nuage condensates to be used as templates for sRNA amplification.

We also suggest that nuage allows for the separation of sRNA amplification mechanisms initiated by primary versus secondary sRNAs (Fig. 3). We propose that, in the cytoplasm of germ cells, only primary sRNA/Argonaute complexes are permitted to initiate sRNA amplification. Secondary Argonautes in the cytoplasm target transcripts for degradation, as in somatic cells. Such a linear pathway guarantees self-extinction of the silencing event when primary sRNAs are no longer present. We propose that secondary Argonautes are permitted to engage with the sRNA amplification machinery only in specialized nuage condensates, such as the Z granules, Mutator foci and SIMR foci. Amplification driven by secondary sRNAs has the potential to be self-perpetuating, since amplified secondary sRNAs can feedback and generate more sRNAs even in the absence of primary sRNAs. Sequestration in nuage of such potentially permanent sRNA amplification loops would ensure that these loops only form on rare transcripts that have first been vetted for sRNA amplification in P granules.

Recent reports have shown that *prg-1* mutants inappropriately generate sRNAs against histone and ribosomal RNAs and exhibit remarkably extended transgenerational inheritance lasting for hundreds of generations (Barucci et al. 2020; Reed et al. 2020; Shukla et al. 2021; Wahba et al. 2021). These observations suggest sRNA amplification mechanisms become dangerously promiscuous in mutants lacking piRNAs, and consistent with this, *prg-1* mutants become completely sterile over successive generations (Barucci et al. 2020; Wahba et al. 2021). Interestingly, in *prg-1* mutants, the secondary Argonaute WAGO-1 mislocalizes to the cytoplasm (Barucci et al. 2020) and late generation *prg-1* mutants frequently lose PGL-1 localization to P granules (Spichal et al. 2021). One possibility, therefore, is that lack of piRNAs/PRG-1 degrades nuage organization over time leading to inappropriate mixing of Argonautes and sRNA amplification machineries in the cytoplasm. Careful analyses comparing protein and RNA localizations in nuage in different genetic contexts will be needed to test these hypotheses further.

## OUTLOOK

We have described how nuage in *C. elegans* is a multicondensate structure that segregates sRNA factors into different compartments. Intriguingly, compartmentalization of nuage has also been reported in other organisms. For example, in the fetal gonocytes of mice, two adjacent nuage domains, pi-bodies, and piP-bodies, contain the piRNA Argonaute proteins MILI, and MIWI2, respectively (Aravin et al. 2008, 2009; Shoji et al. 2009). Similarly, piNG-bodies, nuage-like structures in *Drosophila* testes required for piRNA silencing, contain an outer layer surrounding a core composed of the PIWI Argonaute Ago3 (Kibanov et al. 2011). The nuage-like Balbiani bodies of fish oocytes are also multilayered assemblies (Roovers et al. 2018). Thus, compartmentalization is likely a conserved feature of nuage across metazoans.

We have proposed that nuage condensates restrict sRNA amplification to prevent dangerous amplification loops. *C. elegans* is unique in relying on RNA-dependent RNA polymerases to generate sRNAs, and therefore it may be argued that animals that use different mechanisms to generate sRNAs may not utilize nuage condensates in the same way. A similar restrictive function, however, has been attributed to Yb bodies, nuage-like compartments in the follicle cells of the *Drosophila* ovary. Armitage is an RNA helicase involved in piRNA biogenesis (Ge et al. 2019; Ishizu et al. 2019). Localization of Armitage in the nuage-like Yb bodies increases its RNA-binding specificity to ensure generation of piRNAs from specific piRNA precursors derived from the flamenco piRNA cluster (Ishizu et al. 2019). In the absence of Yb bodies, Armitage binds transcripts promiscuously and aberrantly generates piRNAs from transcripts abundant in the cytoplasm (Ishizu et al. 2019). Nuage compartments serving as “circuit breakers” to prevent promiscuous silencing may therefore be a conserved feature of sRNA silencing pathways. A challenge for the future will be to understand how the liquid-like properties of nuage condensates tune the selectivity of RNA–protein interactions in the sRNA silencing machinery.

## COMPETING INTEREST STATEMENT

G.S. serves on the Scientific Advisory Board of Dewpoint Therapeutics, Inc.
